# *Escherichia coli* SeqA Structures Relocalize Abruptly upon Termination of Origin Sequestration during Multifork DNA Replication

**DOI:** 10.1371/journal.pone.0110575

**Published:** 2014-10-21

**Authors:** Solveig Fossum-Raunehaug, Emily Helgesen, Caroline Stokke, Kirsten Skarstad

**Affiliations:** 1 Department of Cell Biology, Institute for Cancer Research, Oslo University Hospital, the Norwegian Radium Hospital, Oslo, Norway; 2 School of Pharmacy, Faculty of Mathematics and Natural Sciences, University of Oslo, Oslo, Norway; University of Oklahoma, United States of America

## Abstract

The *Escherichia coli* SeqA protein forms complexes with new, hemimethylated DNA behind replication forks and is important for successful replication during rapid growth. Here, *E. coli* cells with two simultaneously replicating chromosomes (multifork DNA replication) and YFP tagged SeqA protein was studied. Fluorescence microscopy showed that in the beginning of the cell cycle cells contained a single focus at midcell. The focus was found to remain relatively immobile at midcell for a period of time equivalent to the duration of origin sequestration. Then, two abrupt relocalization events occurred within 2–6 minutes and resulted in SeqA foci localized at each of the cell’s quarter positions. Imaging of cells containing an additional fluorescent tag in the origin region showed that SeqA colocalizes with the origin region during sequestration. This indicates that the newly replicated DNA of first one chromosome, and then the other, is moved from midcell to the quarter positions. At the same time, origins are released from sequestration. Our results illustrate that newly replicated sister DNA is segregated pairwise to the new locations. This mode of segregation is in principle different from that of slowly growing bacteria where the newly replicated sister DNA is partitioned to separate cell halves and the decatenation of sisters a prerequisite for, and possibly a mechanistic part of, segregation.

## Introduction

DNA replication in the bacterium *Escherichia coli* is initiated at the replication origin, *oriC*, and proceeds bidirectionally towards the terminus, Ter [1]. In slowly growing *E. coli* cells initiation of replication occurs at one origin and the circular chromosome is organized into a dynamic helical ellipsoid [2] with the left and right replichores in separate cell halves before replication [3], [4]. The origin is situated at midcell and the two newly replicated origins stay colocalized there for about 20 minutes [5]–[8]. Following this period of colocalization specific translocation of the sister origins occurs, possibly via the centromere-like *migS* site [9] and/or other unknown mechanisms, to each of the cell halves. Also other chromosomal loci colocalize for about 10 minutes after replication [5], [8], possibly due to intertwining of the DNA before the precatenanes are removed by topoisomerase IV [10]. Transient colocalization of sister chromosomes [6], [11]–[15] and proper chromosome organization by the MukBEF complex [16]–[18] has been shown to be important to ensure proper chromosome segregation.

*E. coli* is a bacterium that grows rapidly and contains several replicating chromosomes in rich medium. Initiation of replication then occurs simultaneously from 2, 4 or 8 origins (the number depending on the growth rate) [19], [20], and sister origins are colocalized during large parts of the cell cycle [7], [11], [21]. For instance, pairs of origins were found to colocalize for an entire generation in cells grown in LB medium [7], [21].

The SeqA protein and the dynamin-like protein, CrfC, have been reported to be involved in colocalization of newly replicated DNA [7], [12], [15], [21]–[23]. The CrfC protein acts on newly replicated DNA in a clamp-dependent manner [22], whereas the SeqA protein binds to newly replicated, hemimethylated GATC sites in the origin region and behind the replication forks [24]–[29]. SeqA was first discovered as a negative regulator of replication initiation [30], [31] that causes origins to be sequestered away from the replication apparatus. Excess SeqA protein was found to prolong the period of origin sequestration and delay separation of newly replicated chromosomes [32]. Recently it was shown that the SeqA protein was required for 20–30 minutes post-replication colocalization of origins and snap sites (sites exhibiting prolonged colocalization) during slow growth [15].

Studies of *E. coli* chromosome segregation have so far indicated movement of fluorescently tagged loci that is either gradual [4], [8], [33], [34] or abrupt [5], [27], [28], [35], and have led to several different segregation models, involving both active and passive segregation mechanisms [14], [36], [37]. In the present work we used fluorescently tagged SeqA protein as a tool to study the dynamic positioning of newly replicated DNA in living cells during rapid growth. We also examined the localization of SeqA structures with respect to FROS (fluorescent-repressor-operator system) -tagged origin and Ter regions. We find that at the end of origin sequestration pairs of newly replicated sister chromosomes move abruptly to the quarter positions.

## Results

### Abrupt relocalization of SeqA bound to newly replicated DNA occurs at the end of origin sequestration

**Figure 1:**
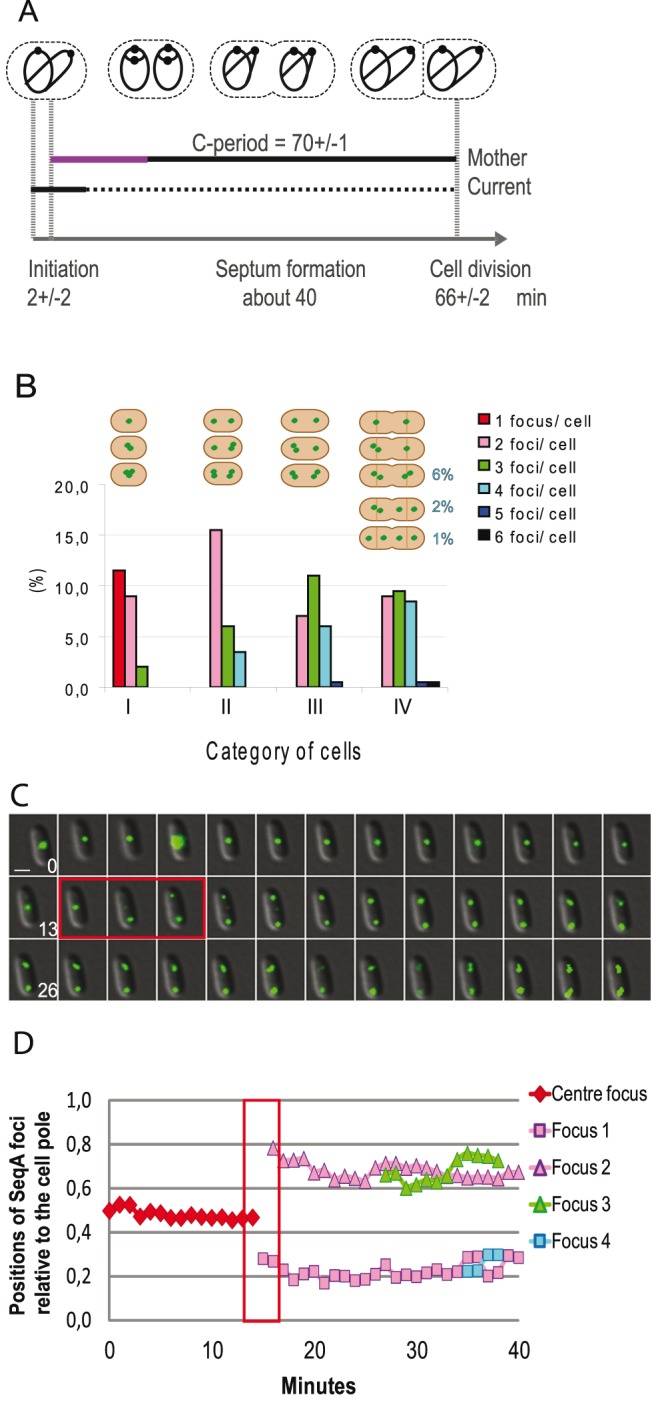
Live-cell fluorescence imaging of SeqA-YFP during rapid growth. Cells with the *YFP* gene inserted at the C-terminal end of the chromosomal *seqA* gene (SF128) were grown at 28°C on an agarose pad containing 1% glucose-CAA. The agarose pad was attached to a microscopy slide, and images were recorded every 1 minute over a 40 minutes period. (**A**) Cell cycle diagram with parameters obtained by flow cytometry (see Figure S1 and Table S1). The replication period (C-period) spanned about one doubling time (τ = 66 min). The origin sequestration period is shown in purple as part of the black line that indicates the C-period. Initiation of replication occurred at age a_i_ = 2 minutes (average for the population). The D-period is shown as a stippled line. Numbers presented in the diagram are average of four independent experiments. Schematic drawings of cells are shown above the diagram to illustrate DNA content, numbers of origins, numbers of replication forks and replication fork progression at different stages of the cell cycle (the drawings are not indicative of chromosome positioning or organization patterns). Chromosomes are shown as black lines and origins as black dots. (**B**) Histogram showing numbers of SeqA foci per cell in categories I–IV representing the progression along the cell cycle (see text for description of categories). Representative illustrations of the cells with SeqA foci (as green dots) within each category are shown above the histogram. (**C**) Time-lapse series of a representative cell from category I with one SeqA focus at midcell. Scale bar is 1 µm. Numbers on pictures indicate time after start of imaging. (**D**) Analysis of SeqA dynamics during live-cell imaging. The positions of the SeqA foci along the cell length were plotted as a function of time for the cell from Figure 1C. The average positions of SeqA foci relative to the cell pole (obtained from six cells from category I) are shown in Figure S3. The red box in (**C**) and (**D**) indicates relocalization of SeqA from midcell towards the quarter positions at 14–16 minutes after start of imaging.

We have investigated segregation patterns of newly replicated DNA through the cell cycle in rapidly growing *E. coli* cells using fluorescently labeled SeqA protein (SeqA-YFP). The SeqA protein binds to hemimethylated GATC sites [24], [38] and colocalizes with new DNA emerging from the replication forks [12], [25]. We performed live-cell imaging every one minute over a 40 minutes period, and obtained the cell cycle parameters by flow cytometry analysis. Cells were grown at 28°C in glucose-CAA medium (τ = 66 min) to early exponential phase (OD ∼0.15), at which time samples were prepared for microscopy or flow cytometry analysis. The period of hemimethylation at *oriC* (sequestration period) was measured by restriction enzyme digestion and found to be similar to that of wild type cells (16+/−1.1 min) (average +/− standard error of the mean (SEM)) (Figure 1A, purple line and Table 1). Flow cytometry analysis showed that initiation of replication occurred simultaneously at all origins present in the cell (initiation synchrony) (Text S1 and Figure S1). This indicated that origin sequestration was not compromised by the YFP-tagging of the SeqA protein. Cell cycle analysis of DNA histograms of cells growing exponentially or treated with rifampicin and cephalexin to allow run-out of replication (Figure S1) indicated that initiation occurred at two origins at an average cell age of 2 minutes (a_i_), and that the replication period (C-period) lasted about 70 minutes (Figure 1A and Table S1). Live-cell imaging showed that SeqA-YFP formed discrete foci within each cell, but these foci started to photo bleach towards the end of the imaging period (see Movie S1 and Figure S2).

**Table 1:** Period of hemimethylation at *oriC*.

Strainname	Fluorescence tag	Fraction of *oriC* inhemimethylated state#	Hemimethylation of*oriC* (min)*
AB1157	None	0.26+/−0.024	20+/−1.2
SF128	*seqA-YFP*	0.30+/−0.033	16+/−1.1
SF131	*seqA-YFP, oriC-CFP*	0.33+/−0.065	17+/−2.8

# determined by restriction digestion of the diagnostic *HphI* site in *oriC* [32], [43].

*calculated by the formula: P_hemimeth_ = –τ ln(2^−ai/τ^–F/2)/ln2–a_i_.

F is the fraction of cells with *oriC* in the hemimethylated state, a_i_ the initiation time and τ the generation time.

# *Values are average +/− standard error of the mean (SEM) of five independent experiments.

Representative cells from live-cell imaging are shown in Figures 1C and S2. The SeqA foci were mostly localized at midcell and the quarter positions (Figure S4A), but the localization pattern throughout the cell cycle was quite complex. Therefore, the cells were categorized (category I to IV) from snapshot images that represent the progression along the cell cycle (Figure 1B and Table 2). In category I we placed small cells with SeqA foci localized at midcell. Most of the cells in category I contained a single SeqA focus and the average cell length was 1.7 µm. We could not determine the exact age of these cells, but since flow cytometry showed that cells initiate at or shortly after cell division (Figure 1A), most of them should contain four replication forks at the start of imaging (t = 0 min). Cells in category II were also relatively small and SeqA foci were localized at the quarter positions. Most of the cells in this category contained two foci and the average cell length was 1.9 µm. Category III included cells of medium size (average cell length was 2.5 µm) with one or two SeqA foci at each quarter position. These cells started to form a septum towards the end of the imaging period. Category IV included cells in which a septum had started to form at t = 0 minutes and average cell length was 2.8 µm. These cells presumably underwent cell division during the imaging period. It was, however, not possible to see exactly at what point cell division occurred because the two newborn cells did not always visibly separate. Figure 1B shows the numbers of SeqA foci per cell within each category, and illustrations of SeqA focus localization within the cells.

**Table 2:** Categories of cells (SF128) with characteristic features from snapshot imaging (Figure 1B).

	Category ofcells	Category ofcells	Category ofcells	Category ofcells
	I	II	III	IV
Average cell length	1.7	1.9	2.5	2.8
(µm)	(1.3–2.2)*	(1.5–2.2)*	(2.3–3.0)*	(2.2–3.2)*
Average numbers ofSeqA foci per cell	1.6	2.6	3.0	3.1

N = 417 cells.

*Minimum and maximum values of cell size within the category.

Figure 1C shows images of a cell from category I with a single midcell SeqA focus at the start of imaging (t = 0 min). The central SeqA focus was occasionally observed as two separate, but closely spaced foci in some cells. This indicated that single midcell foci in reality might represent two separate foci, but that limitations in microscopic resolution hinder visualization of this phenomenon. We found that the midcell SeqA focus remained at its position for 14 minutes, and was then rapidly replaced by foci at the ¼ and ¾ positions of the cell (Figures 1C and D, red squares). The transition of SeqA from midcell to the quarter positions occurred in two steps. First a midcell SeqA focus was replaced by a single focus at one cell quarter and the second focus remained at midcell. Then, within a 2 minutes interval, the midcell focus was replaced by a focus at the opposite cell quarter (Figures 1C and D, red squares). In some cases it was also possible to see a very weak midcell foci that persisted for a few minutes after the relocalization step (Figure 1C, t = 17 min and t = 18 min).

Live-cell imaging of 41 category I cells from four independent experiments indicated that relocalization of SeqA from midcell to the first quarter position occurred on average 11 minutes after start of imaging (t = 0 min) (Table S2). Then 2–6 minutes later, relocalization to the second quarter position occurred (Table S2). Replication was initiated at or shortly after cell division (Figure 1A), thus, the point of SeqA relocalization in the cell’s life coincides with the end of the origin sequestration period (Table 1).

The positioning of SeqA relative to the cell pole over time was also analyzed (SeqA dynamics). For the cell presented in Figure 1C, we found that SeqA was quite immobile at midcell before the relocalization event took place (Figure 1D, red diamonds). Analysis of SeqA positioning at the quarter positions indicated a higher degree of movement compared to that at the midcell position. Towards the end of the imaging period we found that the SeqA foci at the quarter positions sometimes resolved into two foci (Figure 1D). Similar patterns of SeqA positioning were found after analysis of five additional cells (Figure S3).

Cells in category II and III contained one or two closely spaced SeqA foci at each of the quarter positions at the start of imaging (Figure 1B). These foci were localized at the quarter positions throughout the imaging period (40 min) and changes in localization and distance between the SeqA foci were relatively small (Figures S2B and C).

The largest cells (category IV) made up 28% of the cell population (Figure 1B), indicating that a septum started to form at an age of about 40 minutes (Figure 1A). Most cells contained either one or two closely situated foci at the quarter positions. However, in a few cells the foci were localized at the one-eighth positions (Figure 1B). We observed a localization pattern of the foci over time that was quite similar to that of the cells in category II and III (see above). This means that the cells mostly contained alternating two, three or four foci per cell. Towards the end of the imaging period, we observed one or two closely spaced SeqA foci at midcell in each cell half (Figure S2D).

The replication pattern of the cells described here is shown in Figure 1A and is based on stable and reproducible replication patterns found during steady-state growth in culture flasks. The live-cell imaging, however, involves cells that were grown on a glucose-CAA containing agarose slide under the microscope. In this situation oxygen might be limiting, and we therefore investigated whether cell cycle parameters changed with time. To simulate the situation under the microscope, cells from an exponentially growing culture were spread onto a large glucose-CAA containing agarose pad (200×200 mm) and incubated at 28°C beneath a glass plate. Samples were washed off and collected after different periods of incubation (0, 15, 30 and 60 min) and analyzed by flow cytometry. We found that the cell cycle parameters did not change significantly, even after 60 minutes of incubation on the agarose slide (Figure S5). A few minutes delay in cell division seemed to be the only change.

### The origin region and SeqA protein are colocalized during sequestration

The above results indicate that the new DNA behind four replication forks (two on each chromosome) resides in a pair of SeqA structures at midcell in the category I cells. Since SeqA stays bound to the origins throughout the sequestration period it seems likely that the origin DNA may be part of the midcell SeqA structures.

We therefore wished to obtain information about the localization of origin and SeqA foci relative to each other through the cell cycle. The same C-terminal fusion of yellow fluorescent protein to SeqA was used and the cells had their *oriC* region marked with fluorescent *lacI* repressor (LacI-CFP) bound to an array of *lac* operators 15 kb counterclockwise of *oriC* [39]. The cells were grown as above in glucose-CAA medium at 28°C.

**Figure 2:**
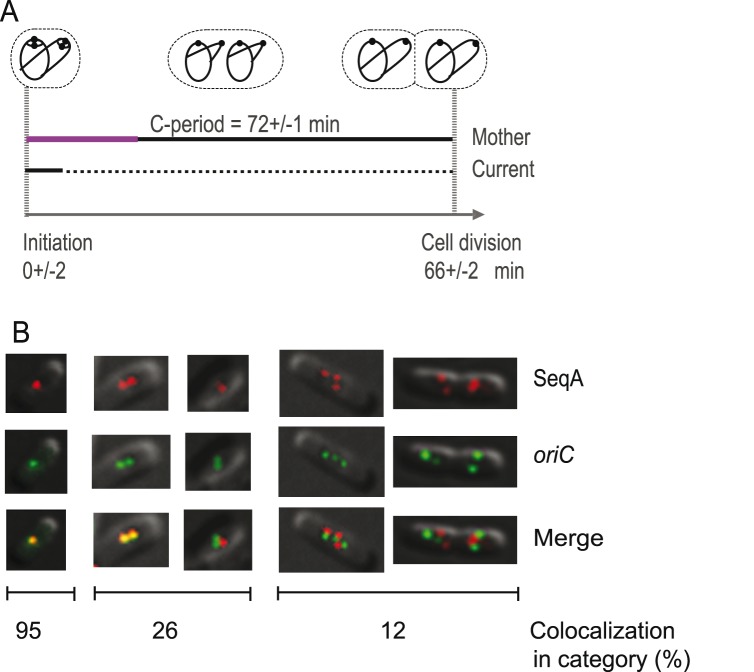
Simultaneous imaging of SeqA and the origin region. (**A**) Cell cycle diagram of cells (SF131), with fluorescently tagged SeqA protein (SeqA-YFP) and origin region (FROS), obtained by flow cytometry (see Figure S1 and Table S1). Cells were grown in glucose-CAA medium with a doubling time (τ) of 66 minutes. Initiation of replication occurred at age a_i_ = 0 minutes (average for the population), and the replication period (C-period) lasted for about 72 minutes. The origin sequestration period is shown in purple as part of the black line that indicates the C-period. The D-period is shown as a stippled line. (**B**) Snapshot fluorescence imaging shows formation of discrete SeqA (pseudo-colored red) and origin (pseudo-colored green) foci. Occurrence of yellow foci indicates colocalization of the SeqA protein and origin region within the resolution of the microscope. The percentage of cells with yellow foci is indicated. N = 476 cells.

Flow cytometry and cell cycle analysis showed that the replication pattern of these cells was quite similar to that of SF128 (compare Figures 1A and 2A). Initiation of replication occurred at cell division (a_i_ = 0 min) and newborn cells therefore had four origins. They replicated with two old forks and four new forks (totally six forks) until termination at age a_t_ = 6 minutes. During the rest of the cell cycle, each cell contained four replication forks (Figure 2A). The period of sequestration of *oriC* was for these cells 17+/−2.8 minutes (average +/−SEM) (Table 1).

Fluorescence microscopy of living cells from the exponentially growing culture (snapshot imaging) revealed the presence of discrete origin and SeqA foci (Figure 2B). The SeqA and origin foci were mainly located at midcell and at the quarter positions, but the localization pattern of the origin foci was somewhat broader (Figure S4B). Analysis of the smallest cells (about 20% of the cell population), containing a single SeqA or origin focus at midcell, indicated colocalization (yellow foci) in 95% of the cells. In contrast, colocalization was observed in only 26% of cells with three or four foci (one SeqA and two origin foci, two SeqA foci and one origin focus or two of each kind per cell) (Figure 2B and Table 3). The largest cells in the population contained two, three or four SeqA or origin foci per cell. We found that the foci in these cells were located to different sub-domains of the cell and that colocalization (presence of yellow foci) was only observed in 12% of the cells (Figure 2B and Table 3). The data presented here suggests that in the smallest cells, SeqA bound to hemimethylated DNA at the replication fork was colocalized with the newly replicated, sequestered origins. This result supports the earlier suggestion that the hemimethylated DNA of sequestered origins is part of, or quite near, the SeqA structure behind the replication forks [40].

**Table 3:** Numbers of cells in which SeqA and origin region are colocalized (yellow foci) or not (red/green foci).

Foci per cell SeqA(red)/*oriC* (green)	Yellow foci(colocalization) (%)	Red/greenfoci (%)	Number ofcells (%)	Average celllength (µm)
1/1	95	5	16	1.7
1/2, 2/1 or 2/2	26	74	61	2.0
2/3, 3/2, 3/3, 4/2,3/4 or 4/3	12	88	19	2.5
4/4	75	25	3	3.0

N = 476 cells.

### The SeqA protein does not colocalize with the Ter region

**Figure 3:**
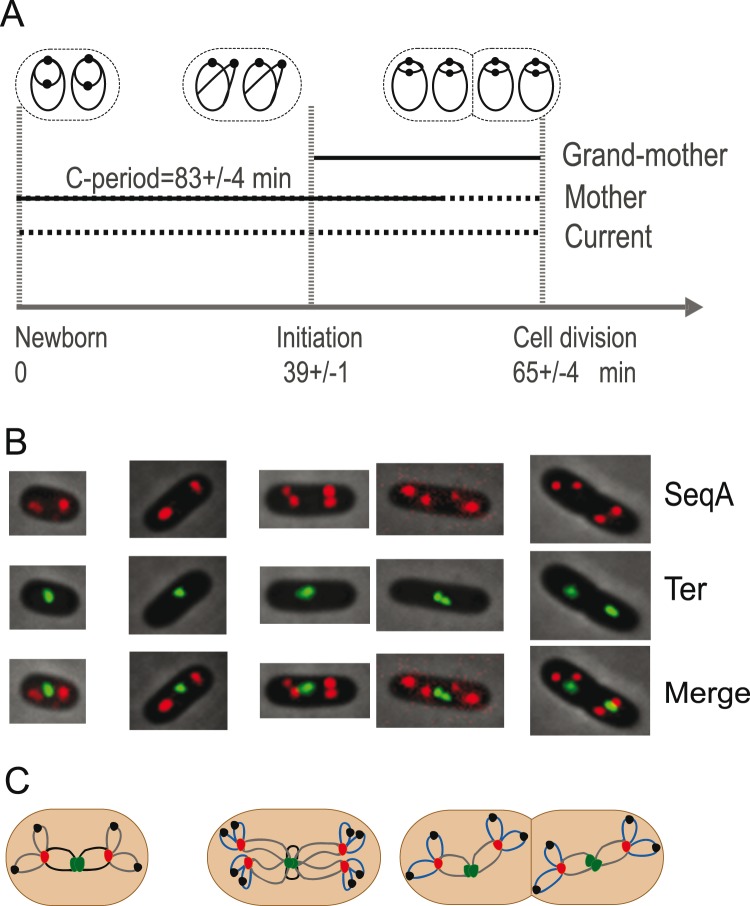
Simultaneous imaging of SeqA and the Ter region. (**A**) Cell cycle diagram of cells (SF163), with tagged SeqA protein (SeqA-YFP) and Ter region (FROS), obtained by flow cytometry (see Figure S1 and Table S1). Cells were grown in glucose-CAA medium with a doubling time (τ) of 65 minutes. Initiation of replication at four origins occurred at age a_i_ = 39 minutes (average for the population), and the replication period (C-period) lasted for about 83 minutes. The C-period is indicated as a black line, and the D-period is shown as a stippled line (**B**) Representative images from snapshot fluorescence imaging shows formation of SeqA(pseudo-colored red) and Ter (pseudo-colored green) foci. N = 232 cells. (**C**) Schematic drawing of DNA and foci below the images, SeqA as red dot, Ter region as green dot, origin as black dot, DNA replicated by old forks in grey, DNA to be replicated by old forks in black and DNA replicated by new forks in blue. The placement of origins and DNA is in this illustration hypothetical and simplified, based on observed positioning of SeqA and Ter region in fluorescence images in addition to cell cycle parameters from 3A.

We also determined to what degree SeqA structures colocalized with the Ter region. We performed snapshot imaging of cells with fluorescently tagged SeqA protein and with a FROS near the Ter region (SF163). The cells were grown as above in glucose-CAA medium at 28°C. Flow cytometry and cell cycle analysis showed that initiation of replication occurred about halfway through the cell cycle, and that the cells contained four, twelve or eight replication forks and two or four Ter regions (Figure 3A). The replication and post replication periods (C and D) were found to be longer in these cells (SF163) compared to SF128 and SF131. Thus, the sequestration period occurred in middle-aged to old cells with SeqA foci localized at the quarter positions.

Fluorescence imaging showed formation of two, three or four SeqA foci that were mainly located at the quarter positions of the cell. The tagged Ter regions were found at midcell as one or two closely spaced foci for most of the cell cycle, but relocalized to the quarter positions before cell division (Figures 3B and S4C). This result indicates that the Ter regions from two chromosomes were colocalized throughout the cell cycle, i.e. separate Ter regions could not be resolved within the resolution of the microscope. This is in accordance with earlier published data [7], [41], [42]. The SeqA and Ter foci did not colocalize (yellow foci not present). However, a few cells (4% of the population) had Ter and SeqA foci that were near each other or overlapped partly. These probably represent the cells in which replication forks were travelling across the Ter-FROS DNA.

## Discussion

### Abrupt relocalization of SeqA structures at the end of origin sequestration

**Figure 4:**
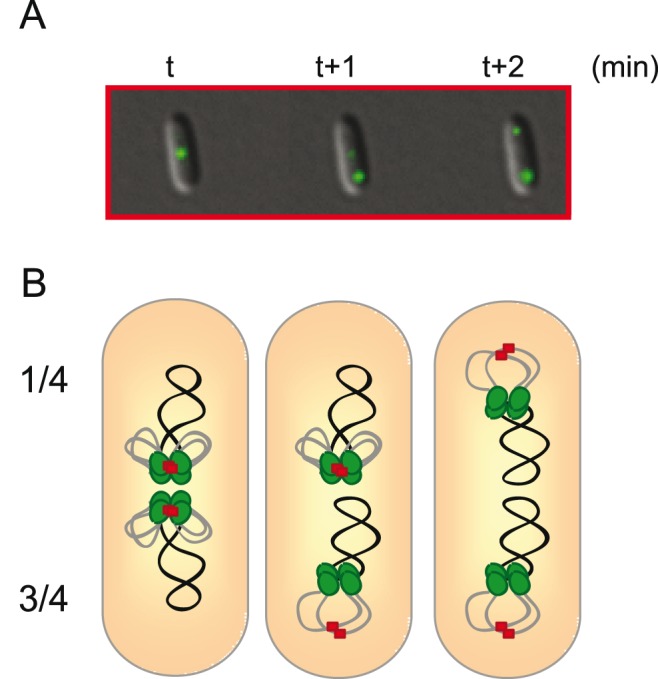
Model of SeqA relocalization events. A schematic drawing to illustrate how new and unreplicated DNA may change places during the SeqA relocalization events reported in Figure 1. (**A**) Images in red square (from Figure 1C) showing SeqA at the end of the period at midcell (t min), the first relocalization to one cell quarter (at t+1 min), and the second relocalization to the other cell quarter (at t+2 min). (**B**) Illustration of the position of SeqA and newly replicated DNA of two chromosomes during the period at midcell (cell number one), after the movement of first new and unreplicated DNA of the lower chromosome (cell number two), and then the upper chromosome (cell number three). It is here suggested that origins in sequestration are part of the midcell SeqA structures. The SeqA foci are illustrated as green dots, the origin foci as red squares, unreplicated DNA as a black line and newly replicated DNA as a grey line. The position of the unreplicated DNA is simplified and the Ter region localization at midcell is not included.

Cells with SeqA-YFP grown at 28°C in glucose-CAA medium were found to initiate replication at two origins early in the cell cycle (when cells were on average 2 minutes old). Live-cell imaging of the smallest cells for a period of 40 minutes showed that SeqA structures complexed with new DNA from the four replication forks were situated at midcell and remained there for about 15 minutes. After this followed a rapid sequence of events which took place within 2–6 minutes. The midcell SeqA focus was replaced by two foci, one at each cell quarter. This relocalization of SeqA foci occurred in two steps, first one SeqA focus appeared at the cell quarter position and then a second SeqA focus appeared at the other quarter position (Figure 4A). We suggest that the appearance of SeqA foci at the quarter positions represents the segregation and rearrangement of new DNA of first one chromosome and then the other. The cartoon in Figure 4B is meant to illustrate how new and old DNA may change places and also that the origins may be released from sequestration at the same moment. Note that the drawing of the old DNA is simplified in the cartoon, omitting information about the positions of the two Ter sites.

The origin sequestration period lasted about 16 minutes, which is in accordance with previous measurements [32], [43], and coincided with the period of midcell SeqA localization. After sequestration origins are freed from SeqA binding and become fully methylated as Dam methylase gains access. It is not known whether SeqA protein is actively removed from origins to bring about the end of sequestration or whether this is a passive process driven by dynamic binding of SeqA to more recently replicated hemimethylated sites. The role of Dam methylase in terminating sequestration is also not clear. It is possible that Dam methylase is hindered from methylating GATC sites by the nature of the SeqA structure and only has access to GATC sites that have escaped from the structure. Since we find that the end of sequestration coincides with the relocalization of SeqA structures, it is possible that the origins escape from the SeqA structures because of the DNA relocalization (see illustration Figure 4B). It might therefore be that the end of sequestration is brought about by this relocalization event. This idea assumes that the SeqA multimer has a passive role and simply follows the new DNA or breaks up and reforms as the DNA is moved, and that the *oriC* DNA is then lost from the structure and becomes fully methylated as Dam methylase gains access. Alternatively, the end of origin sequestration may be an active process which frees the tethered origins and allows the relocalization event to occur. It could be that sequestration of origins involves a component that causes tethering to the membrane. Hemimethylated *oriC* DNA has been found in membrane fractions and it has been suggested that origin sequestration is localized to the inner or outer membrane, or to areas where the two membranes are fused [38], [44]. It might also be possible that the SeqA multimer bound to new DNA has a more active role in chromosome segregation, possibly by interacting with so far uncharacterized segregation proteins.

### Newly replicated sister loci are partitioned to separate cell halves during slow growth but not during rapid growth

The present growth situation where the cell has two replicating chromosomes, with two forks on each chromosome throughout the cell cycle, is one of the simplest and was chosen for this reason. The results show that one chromosome experiences abrupt segregation of both sisters of the newly replicated DNA 2–6 minutes before the other chromosome. The reason for this lack of simultaneousness remains to be investigated, but could occur if the two chromosomes are acted upon independently, i.e. that movement within one cell half occurs independently of movement within the other cell half.

During slow growth sister strand cohesion has been found to be promoted by the SeqA protein [15] and *in vitro* SeqA has been found to cause cohesion of newly replicated sisters [21]. Regulation of the activity of topoisomerase IV has been suggested to be involved in the SeqA dependent cohesion [15], and the actual cohesion might thus be caused by delayed removal of precatenanes. The prolonged cohesion of sister chromosomes at so-called snap loci has been suggested to lead to a build-up of tension [2], [6]. When the tension becomes too great, abrupt separation of the sister chromosomes occurs [15]. Although a build-up of tension may be a reason for the abruptness of movement of the new DNA of each of the two chromosomes described here, cohesion of sisters and separation of sister chromosomes cannot have a role. This is because the two newly replicated sisters on each chromosome co-segregate. Thus, the main difference in the situation we describe here compared to the situation in slowly growing cells is that the new DNA behind the two replication forks and the two copies of the origin region on a replicating chromosome are moved together. This makes sense since it is the two “old” chromosomes that must be separate at division and not the “new” pairs of sisters on each of these chromosomes.

### SeqA bound to new DNA is immobile at midcell

As mentioned, all the new DNA bound by SeqA was situated at the cell center in the category I cells and analysis of SeqA dynamics indicated a relatively high degree of immobility. The reason for the immobility of the midcell SeqA focus could be that the SeqA structures trailing the forks are physically part of the SeqA structures at *oriC*. It seems likely that the *oriC*-SeqA structures are part of the SeqA structures trailing the replication forks because the GATC-dense origins would be part of the SeqA structures ever since they were formed at the beginning of sequestration. This idea is built on the finding that GATC-dense DNA stays bound to SeqA longer than GATC-sparse DNA [25], [45].

The period of SeqA immobility at midcell also coincides with the period in which the Ori macrodomain is replicated [3], [42]. The Ori macrodomain is a large region of about 1 Mb adjacent to *oriC* on the right replichore (about 80 to 100 minutes) and would in our experiments be replicated in about 14 minutes. The region has been shown to exhibit a high degree of colocalization/cohesion [6], [42]. The reason for this could be the extra tethering of the DNA caused by the combined origin-replication fork structure generated by SeqA.

After the relocalization event a very weak SeqA focus could still be distinguished at midcell. It is reasonable to assume that this focus is real and represents a small fraction of the SeqA protein in the cell. It is, however, not clear whether this SeqA is bound to hemimethylated DNA or not. Since the replication cycles of these cells do not overlap very much (see Figure 1A) it is unlikely that the weak focus represents SeqA trailing the old replication forks. This is also unlikely for a second reason, and that is because the Ter domain contains few clusters of GATC sites to which the SeqA protein prefers to bind [25], [45]. As mentioned above, it has been suggested that SeqA structures are anchored in the membrane [38], [44]. Thus, it could be that the observed weak focus represents remnants of SeqA at a membrane site. Irrespective of whether SeqA-*oriC* structures are anchored at the membrane or not, the weak focus seems to indicate that the process of relocalizing SeqA involves disassembly and reassembly of SeqA structures.

### SeqA is bound at pairs of replication forks after the relocalization event

As discussed above, live-cell imaging of SeqA showed that after the relocalization event the new DNA from one chromosome’s two forks was localized at the cell’s quarter position and the new DNA of the other chromosome’s two forks at the three-quarter position. The *E. coli* SMC proteins MukBEF have been shown to be positioned at the quarter positions of the cell early in the cell cycle during rapid growth [7] and may be important for the relocalization event. However, there is so far no evidence that SeqA interacts with MukBEF complexes. It has been shown that topoisomerase IV (TopoIV) interacts with the hinge region of MukB [46]–[48] and since SeqA affects the activity of TopoIV [15], [49] it is possible that the MukBEF complexes at the quarter positions are indirectly involved in the segregation of new DNA to the quarter positions. However, as mentioned, the possible involvement of TopoIV would not be the same as during slow growth. It has been shown that MukBEF plays an important role in segregation of origins [50]. Thus, it is also possible that the MukBEF complexes at the quarter positions interact with the origins that have been freed from sequestration.

After the relocalization event, one SeqA focus at each quarter position was observed. Then, when the replication forks had traveled through the left and right macrodomains, i.e. further than halfway, the two quarter position foci often were seen as four (Figures 1 and S2). This means that the SeqA structures behind the leftgoing and rightgoing replisomes were far enough apart to be distinguished. Analysis of SeqA dynamics at the quarter positions indicated a higher degree of mobility compared to at the midcell position. The reason for this could be that the SeqA structures now trail the replisome which tracks along the parent DNA [51], and are no longer tethered by binding to the *oriC*-SeqA structure.

### Colocalization of Ter regions occurs in a SeqA independent manner

Towards the end of replication when the forks are near, or have entered, the Ter domain, only two midcell SeqA foci were observed (Figure S2D). The reason for this might be that the Ter domain DNA is confined to a limited space. Most of these SeqA foci did not colocalize with the FROS-tagged Ter, probably because this region has few GATC sites and therefore the DNA will not be included in SeqA structures. The result is in agreement with recent ChIP-Chip analysis showing low binding of SeqA in the region around Ter [25].

We found that two Ter regions were colocalized throughout the cell cycle in accordance with earlier published data [7], [41], [42]. Since the SeqA protein did not colocalize with Ter, the result indicates that colocalization of the two Ter copies occurs independently of SeqA. This is accordance with the suggestion that MatP may be the main organizer of the Ter macrodomain [52].

## Materials and Methods

### Bacterial Strains

All strains used are derivatives of the *E. coli* K-12 strain AB1157 [53]. Localization studies of SeqA were done with cells containing YFP fused to the C-terminal end of SeqA. The *seqA-yfp* gene fusion replaced the wild type *seqA* gene at the *seqA* locus and was expressed from its endogenous chromosomal promoter. The YFP protein (also called Venus) was from [54] and connected to SeqA via a four amino acid linker [55].

The YFP protein used in this study was found to fold well and mature efficiently [54]. It contains the following mutations: F46L, S65G, V68A, S72A, S175G and T203Y [54], [56] and is a weak dimer. We found that the SeqA-YFP fusion was functional in origin sequestration (Table 1) and western blot analysis showed that the cellular concentration of fluorescently tagged SeqA was about the same as that of wild-type SeqA (data not shown).

We have also studied cells containing SeqA fused to other GFP variants: YPet, mKate2 and EGFP. We found that, for these cells, origin sequestration was compromised and initiation of replication did not occur simultaneously at all origins present within a single cell (unpublished results). Fluorescence imaging of all the different SeqA-fusion proteins showed the presence of discrete SeqA foci within the cells (Figure 1 and data not shown). However, only cells with SeqA-YFP contained distributions of SeqA foci that were essentially the same as those obtained with immunofluorescence microscopy [40]. The reason why the SeqA-YFP fusion is fully functional and not the other SeqA fusions remains to be investigated. SeqA-YFP was transferred into AB1157 by P1[57] transduction to obtain SF128.

FROS (fluorescent-repressor-operator system) was used to study the localization of the origin and terminus regions [39]. To obtain SF131 a *lac* operator array located at the *att*Tn7 site (at 84.27 min) 15 kb counterclockwise from *oriC* (from IL01) [39], [58] was P1 [57] transduced into SF128 cells. To obtain SF163 a *tet* operator array located 50 kb clockwise from *dif* (*ter3*) (from IL04) [39], [58] was P1 transduced [57] into SF128 cells. The SF131 and SF163 strains also contained an expression vector containing *lac* repressor fused to cyan fluorescent protein (LacI-CFP) or *tet* repressor fused to cyan fluorescent protein (TetR-CFP) [39], respectively.

### Cell Growth

Cells were grown at 28°C in AB minimal medium [59] supplemented with 1 µg ml^−1^ thiamine, 0.2% glucose and 0.5% casamino acid (glucose-CAA medium). Cells were grown to OD ∼0.15 (exponential phase) at which time they were prepared for flow cytometry analysis or fluorescence microscopy. Expression of fluorescence protein fused LacI or TetR on plasmid was induced at an optical density of 0.04 with arabinose (0.05%) in the presence of IPTG (1 mM) or arabinose (0.02%) in the presence of anhydrotetracycline (80 ng ml^−1^), respectively [39], [58].

### Flow cytometry and cell cycle analysis

Exponentially growing cells (OD ∼0.15) were fixed in ethanol (exponential samples) or treated with 300 µg/ml rifampicin and 10 µg/ml cephalexin for 3–4 hours prior to fixation (run-out samples). Rifampicin inhibits replication initiation [19] and cephalexin inhibits cell division [60]. The cells therefore ended up with an integral number of chromosomes [19], which represents the number of origins at the time of drug treatment (replication run-out). Flow cytometry analysis was performed as previously described [61] using a LSR II flow cytometer (BD Biosciences). The data obtained from the flow cytometry measurement was analyzed by FlowJo 7.2.5 software.

The cell cycle parameters (initiation age (a_i_), C- and D-periods) were calculated by combining the data from the flow cytometry analysis with the theoretical age distribution of an exponential culture and the generation time obtained by OD measurements in an excel based simulation program [62].

### Determination of the sequestration period

Cells were grown in glucose-CAA medium at 28°C and chromosomal DNA was isolated from an exponentially growing culture (OD ∼0.15). Chromosomal DNA was then digested with *XhoI* and *HphI* and electrophoresed on a 1.7% agarose gel. The restriction enzyme *HphI* is methylation sensitive (its recognition site (GGTGA) overlaps half of a GATC) and will not cut when the DNA is methylated. Therefore, when the DNA is fully methylated, enzymatic digestion yields a 492 base pair *oriC* fragment (uncut), whereas unmethylated DNA yields a 234 base pair *oriC* fragment (cut) [32], [43]. Unmethylated chromosomal DNA from *dam* ^−^ cells (JM110) was included as a control. The DNA in the gel was neutralized, transferred to a nylon membrane (Hybond N^+^ Membrane, GE Healthcare Life Sciences) and hybridized to an [α-^32^P]dCTP labelled *oriC* probe [32]. The membrane was scanned on a Pharos FX Plus and quantified using Quantity One software (both from BioRad).

### Widefield microscopy

For widefield live-cell or snapshot fluorescence imaging exponentially growing cells (OD ∼0.15) were immobilized on a 17×28 mm agarose pad (1% in glucose-CAA medium or phosphate-buffered saline, respectively). During live-cell imaging, one to three images were acquired before the 0 minute image and are in this work called snapshot images (see Figures 1B and S4). Fluorescence and differential interference contrast (DIC) images were acquired with a Leica DM6000 microscope equipped with a Leica DFC350 FX monochrome CCD camera and a Plan Apochromat 100X/1.46 N.A objective. Fluorescence and phase-contrast images (for image analysis with MicrobeTracker) were acquired with a HCX PLAPO 63x/1.40 NA objective. Narrow band-pass filter sets (CFP: Ex BP436/20, Em BP480/40, YFP: Ex BP510/20, Em BP560/40) were used for fluorescence imaging. For multicolor imaging colocalization was calibrated using TetraSpeck Microspheres (0.2 and 0.5 µm) (Molecular Probes). Images were processed and analyzed by MBF Image J, Adobe Photoshop CS4 software. Analysis of cell length and the position of fluorescent foci relative to the cell pole were performed with the MATLAB-based software MicrobeTracker [63].

The time at which the septum started to form (a_s_) was calculated from the formula F = 1–[2–2^(τ–as)/τ^]. F is the fraction of cells in which the septum had started to form as obtained with differential interference contrast (DIC) microscopy.

## Supporting Information

Figure S1 **Flow cytometry analysis of AB1157 and derivatives.** Cells were grown at 28°C in glucose-CAA medium to OD ∼0.15 and samples were subjected to flow cytometry. Experimental DNA histograms (of one representative experiment) of exponentially growing cells are shown in left panels and rifampicin and cephalexin treated cells are shown in right panels. The red lines represent the theoretical simulation with best fit to the data obtained from exponentially growing cells (left panels) [4]. A simulation was performed for each experiment and the C and D values giving the best fit recorded. An average was calculated for each strain (Table S1). The resulting replication pattern diagrams based on at least three independent experiments are shown in Figures 1A, 2A and 3A.
(EPS)

Figure S2 **Time-lapse series of cells containing SeqA-YFP.** Time-lapse series from live-cell imaging of representative SeqA-YFP tagged cells (SF128) from category I to IV representing the progression along the cell cycle (see main text for description of categories). **(A)** Category I, **(B)** category II, **(C)** category III and **(D)** category IV. The YFP fluorescent signals are reported in green. The series shown in (A) is the same as shown in Figure 1C. Bar is 1 µm.
(PDF)

Figure S3 **Analysis of SeqA dynamics during live-cell imaging.** Analysis of the positions of SeqA foci relative to the cell pole throughout the imaging period (40 min) of six cells (SF128) from category I. Data are collected from two independent live-cell imaging experiments. The SeqA foci remained relatively immobile at midcell (Centre focus, red diamonds). On the other hand, when SeqA foci were localized at the quarter position the positions, we observed a higher degree of movement (Foci 1–4). Error bars represent standard deviation.
(EPS)

Figure S4 **Analysis of the position of fluorescent foci relative to cell pole.** Analysis of cell length and the position of fluorescent foci relative to the cell pole using widefield snapshot microscopy and MATLAB-based software MicrobeTracker [5]. The cell outline was obtained with the cell meshes tool of phase-contrast images whereas foci were detected using the SpotFinderZ tool of fluorescent images. The parameters were trained for each set of images. **(A)** Cells with YFP-tagged SeqA protein (SF128), **(B)** cells with YFP-tagged SeqA protein/CFP-tagged *oriC* region (SF131) and **(C)** cells with YFP-tagged SeqA protein/CFP-tagged Ter region (SF163).
(EPS)

Figure S5 **Flow cytometry analysis of cells grown on a microscope slide.** SeqA-YFP tagged cells (SF128) were grown in glucose-CAA medium to OD ∼0.15. Then, 25 ml culture was harvested, resuspended in 1 ml of the same medium and spread on a 200×200 mm agarose slide. The cells were covered with a thin glass plate and incubation was continued at 28°C. After 0, 15, 30 and 60 min, the cells were washed off with TE buffer and prepared for flow cytometry (see above). Analysis of exponential (left panels) and rifampicin/cephalexin treated (right panels) cells showed that the replication pattern did not change significantly over time. The main change seemed to be a few minutes delay in cell division.
(EPS)

Table S1 **Cell cycle parameters of cells grown in glucose-CAA medium at 28°C.**
(DOCX)

Table S2 **Analysis of SeqA relocalization from midcell to the quarter positions during live-cell imaging of SeqA-YFP tagged cells (SF128).**
(DOCX)

Text S1 **Flow cytometry and cell cycle analysis, microscopy sample preparation and investigation of growth on a microscopy slide.**
(DOCX)

Movie S1 **Movie of cells containing SeqA-YFP.** Movie of SeqA-YFP tagged cells (SF128) from live-cell imaging. Images were acquired every one minute. The YFP fluorescent signals are reported in green.
(WMV)
