# Supplementary figures and images for: Escherichia coli SeqA Structures Relocalize Abruptly upon Termination of Origin Sequestration during Multifork DNA Replication
Source: PLoS One. 2014 Oct 21;9(10):e110575. doi: 10.1371/journal.pone.0110575 (PMC4204900; doi:10.1371/journal.pone.0110575)

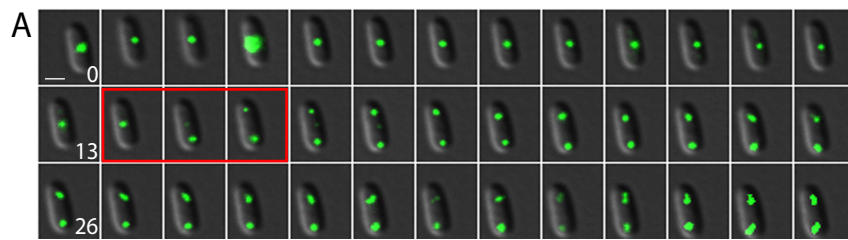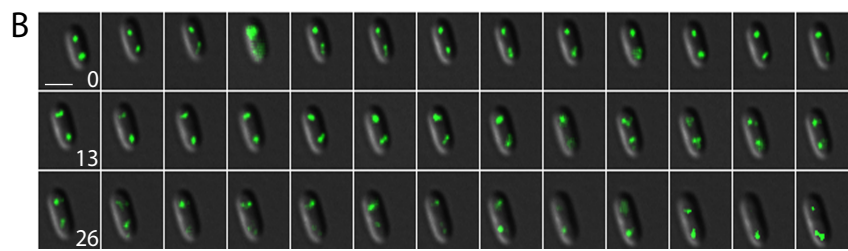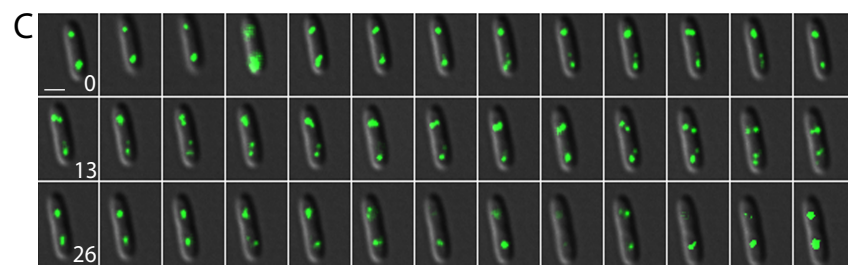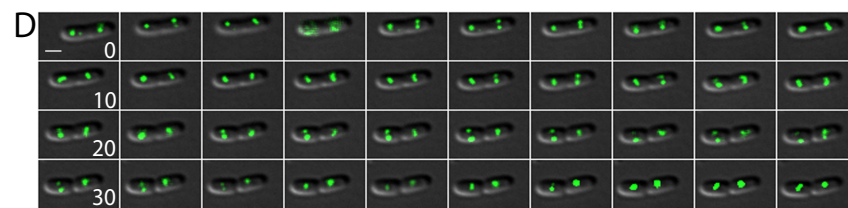

Supplement: Figure S2 — Time-lapse series of cells containing SeqA-YFP. Time-lapse series from live-cell imaging of representative SeqA-YFP tagged cells (SF128) from category I to IV representing the progression along the cell cycle (see main text for description of categories). (A) Category I, (B) category II, (C) category III and (D) category IV. The YFP fluorescent signals are reported in green. The series shown in (A) is the same as shown in Figure 1C. Bar is 1 µm. (PDF) [file pone.0110575.s002.pdf]
