# Supplementary material for: Escherichia coli SeqA Structures Relocalize Abruptly upon Termination of Origin Sequestration during Multifork DNA Replication
Source: PLoS One. 2014 Oct 21;9(10):e110575. doi: 10.1371/journal.pone.0110575 (PMC4204900; doi:10.1371/journal.pone.0110575)
Supplement: Table S1 — Cell cycle parameters of cells grown in glucose-CAA medium at 28°C. (DOCX) [file pone.0110575.s006.docx]

**Table S1 Cell cycle parameters of cells grown in glucose-CAA medium at 28^o^C.**

| **Strain** | **Fluorescence marker** | **τ**  **(min)** | **C^*^**  **(min)** | **C+D^†^**  **(min)** | **Initiation age (a_i_)^†^**  **(min)** |
| --- | --- | --- | --- | --- | --- |
| AB1157 | Wild-type | 61+/-5 | 81+/-6 | 146+/-14 | 36+/-7 |
| SF128 | SeqA | 66+/-2 | 70+/-1 | 126+/-6 | 2+/-2 |
| SF131 | Origin and SeqA | 66+/-2 | 72+/-1 | 129+/-6 | 0+/-2 |
| SF163 | SeqA and Ter | 65+/-4 | 83+/-4 | 156+/-11 | 39+/-1 |

* Determined by combining the data from the flow cytometry analysis, the theoretical age distribution of an exponential culture and the generation time obtained by OD measurements in an excel based simulation program [4]

^†^ Determined by analyzing rifampicin run-out DNA histograms obtained from flow cytometry (see above).

The values are average +/- standard deviation of at least three independent experiments.
