# Supplementary material for: Escherichia coli SeqA Structures Relocalize Abruptly upon Termination of Origin Sequestration during Multifork DNA Replication
Source: PLoS One. 2014 Oct 21;9(10):e110575. doi: 10.1371/journal.pone.0110575 (PMC4204900; doi:10.1371/journal.pone.0110575)
Supplement: Table S2 — Analysis of SeqA relocalization from midcell to the quarter positions during live-cell imaging of SeqA-YFP tagged cells (SF128). (DOCX) [file pone.0110575.s007.docx]

**Table S2 Analysis of SeqA relocalization from midcell to the quarter positions during live-cell imaging of SeqA-YFP tagged cells (SF128).**

| **Experiment** | **SeqA relocalization to first quarter position**  **(min)^*^** | **SeqA relocalization to second quarter position**  **(min)^*^** | **Number of cells** |
| --- | --- | --- | --- |
| 1^#^ | 5+/-0.0 | 11+/-1.7 | 5 |
| 2^#^ | 10+/-1.7 | 13+/-1.3 | 19 |
| 3^#^ | 10+/-1.3 | 15+/-1.3 | 10 |
| 4^#^ | 17+/-1.9 | 19+/-1.4 | 6 |
| **Mean (experiment 1-4)** ^¤^ | **11+/-1.2** | **14**+/-**1.4** | **40** |

* Time indicated represents minutes after start of imaging newborn cell.

^#^ Average +/- standard error of the mean (SEM) within experiment

^#^ Average +/- standard error of the mean (SEM) experiment 1-4
