# Supplementary material for: Escherichia coli SeqA Structures Relocalize Abruptly upon Termination of Origin Sequestration during Multifork DNA Replication
Source: PLoS One. 2014 Oct 21;9(10):e110575. doi: 10.1371/journal.pone.0110575 (PMC4204900; doi:10.1371/journal.pone.0110575)
Supplement: Text S1 — Flow cytometry and cell cycle analysis, microscopy sample preparation and investigation of growth on a microscopy slide. (DOCX) [file pone.0110575.s008.docx]

## Supporting Information

### Flow cytometry and cell cycle analysis

Exponentially growing cells were harvested and fixed (exponential sample) or treated with 300 µg ml^-1^ rifampicin and 10 µg ml^-1^ cephalexin for three to four generations before fixation (run-out sample). Rifampicin inhibits replication initiation [1] and cephalexin inhibits cell division [2] allowing ongoing replication to finish. The cells ended up with an integral number of chromosomes [1], which represents the number of origins at the time of drug treatment. In a culture of cells with synchronous initiation, the integral number of chromosomes is 2^n^ (*n* = 0, 1, 2…). Asynchronous initiation results in cells with an integral number of chromosomes different from 2^n^.

Flow cytometry analysis was performed as previously described [3] using a LSR II flow cytometer (BD Biosciences). The data obtained from the flow cytometry was analyzed by FlowJo 7.2.5 software and used to determine cell cycle parameters in an excel based simulation program [4].

### Microscopy

Growth was performed in glucose-CAA medium at 28^o^C and exponentially growing cells were harvested at OD ~ 0.15. Cells were concentrated and were immobilized on a 17x28 mm agarose pad, 1% in glucose-CAA medium for live-cell imaging or phosphate-buffered saline for snapshot imaging. For live-cell imaging growth was then continued at 28^o^C beneath the microscope and pictures were recorded every 1 min over a 40 min period. During live-cell imaging, one to three images were acquired before the 0 min image and are in this work also called snapshot images. Samples for flow cytometry and cell cycle analysis were prepared from the same culture (see above).

Four independent live-cell experiments were performed. Representative cells from Experiment 4 are shown in Movie S1 and Figures 1C and S2A-D. Table S2 shows data obtained from analysis of the time (in min) at which the SeqA protein was situated at midcell (mid column) and SeqA localized at the quarter positions of the cell (right column) after the relocalization event. The average initiation age (a_i_) in Experiment 1-4 was 0, 1, 0 and 6 min, respectively.

**Investigation of growth on a microscope slide**

Live-cell imaging involved growth of cells on an agarose slide under the microscope. In this situation oxygen might be limiting and we investigated with flow cytometry whether cell-cycle parameters would change with time (Figure S5).

Reference List

1. Skarstad K, Boye E, Steen HB (1986) Timing of initiation of chromosome replication in individual *Escherichia coli* cells. EMBO J 5: 1711-1717.

2. Boye E, Løbner-Olesen A (1991) Bacterial growth control studied by flow cytometry. Res Microbiol 142: 131-135.

3. Torheim NK, Boye E, Løbner-Olesen A, Stokke T, Skarstad K (2000) The *Escherichia coli* SeqA protein destabilizes mutant DnaA204 protein. Mol Microbiol 37: 629-638.

4. Stokke C, Flatten I, Skarstad K (2012) An easy-to-use simulation program demonstrates variations in bacterial cell cycle parameters depending on medium and temperature. PLoS One 7: e30981.

5. Sliusarenko O, Heinritz J, Emonet T, Jacobs-Wagner C (2011) High-throughput, subpixel precision analysis of bacterial morphogenesis and intracellular spatio-temporal dynamics. Mol Microbiol 80: 612-627.
